# Synergistic Effects of Plant Growth Regulators and Elicitors on α-Humulene and Zerumbone Production in *Zingiber zerumbet* Smith Adventitious Root Cultures

**DOI:** 10.3390/molecules27154744

**Published:** 2022-07-25

**Authors:** Nurul Huda Alwakil, Mohamad Suffian Mohamad Annuar, Mahanom Jalil

**Affiliations:** 1 Institute of Biological Sciences, Faculty of Science, Universiti Malaya, Kuala Lumpur 50603, Malaysia; nalwakil12@um.edu.my (N.H.A.); suffian_annuar@um.edu.my (M.S.M.A.); 2 Centre of Biotechnology for Agriculture Research (CEBAR), Universiti Malaya, Kuala Lumpur 50603, Malaysia; 3 Centre for Foundation Studies in Science, Universiti Malaya, Kuala Lumpur 50603, Malaysia

**Keywords:** phytomedicinal, secondary metabolites, in vitro, methyl jasmonate, salicylic acid

## Abstract

*Zingiber zerumbet*, also known as ‘Lempoyang’, possesses various phytomedicinal properties, such as anticancer, antimicrobial, anti-inflammatory, antiulcer, and antioxidant properties. Secondary metabolites possessing such properties i.e., zerumbone and α-humulene, are found dominantly in the plant rhizome. Synergistic effects of plant growth hormones and elicitors on in vitro α-humulene and zerumbone production, and biomass growth, in adventitious root culture (AdRC) of *Z. zerumbet* cultivated in a two-stage culture are reported. The culture was induced by supplementation of 1.0 mg/L NAA and 2.0 mg/L IBA (dark), and subsequently maintained in medium supplemented with 1 mg/L NAA and 3 mg/L BAP (16:08 light-dark cycle), yielded the production of zerumbone at 3440 ± 168 µg/g and α-humulene at 3759 ± 798 µg/g. Synergistic elicitation by 400 μM methyl jasmonate (MeJa) and 400 μM salicylic acid (SA) resulted in a 13-fold increase in zerumbone (43,000 ± 200 µg/g), while 400 μM MeJa and 600 μM SA produced a 4.3-fold increase in α-humulene (15,800 ± 5100 µg/g) compared to control.

## 1. Introduction

The family Zingiberaceae includes various species, such as *Kaempferia galanga* L. [1], *Curcuma longa* [2], *Elettaria cardamomum* Maton. [3], *Zingiber officinale* [4], and *Zingiber zerumbet* [5], that possess medicinal properties. *Zingiber zerumbet* Smith, commonly known as Lempoyang, is a small, perennial, medicinal herb plant that has been established and cultivated mainly in Asia and subtropical regions [6]. Its rhizome extract consists primarily of zerumbone (37%), followed by α-humulene (14%) and camphene (13.8%), which have been shown to exhibit important pharmacological properties, such as anti-inflammatory [7], anticancer, antibacterial, antipyretic [8], anti-allergic, anti-hyperglycemic, anti-ulcer, antioxidant, and antimicrobial properties [9]. Hence, the rhizome has been extensively cultivated and investigated for bioactive compounds. However, *Z. zerumbet* plant mass could be stored only for a short duration due to fungal diseases that deteriorate the rhizome [9]. Furthermore, the quality and quantity of the secondary metabolites are adversely affected by the application of pesticides, climate change, and diseases [10]. Despite plant cell culture technology, the production of targeted bioactive compounds using cell suspension culture is challenging due to the high water content in cells, continuous foaming in the bioreactor, and the unstable production of metabolites [11]. Besides, only a small number of secondary metabolites were found in cell suspension cultures of *Z. zerumbet* [5].

Alternatively, adventitious root culture (AdRC) could serve as natural biofactories that grow vigorously in a formulated media with great potential to produce beneficial plant-derived compounds. The formation of the adventitious root is a complex process influenced by endogenous- and exogenous factors [12]. One of the important factors is plant growth regulators (PGRs), which play a critical role in biomass growth and secondary metabolite production [13]. Generally, a high auxin-to-cytokinin ratio favors root formation, while a high cytokinin-to-auxin ratio favors shoot formation [14]. There are many examples of pharmaceutically important medicinal plants in which AdRC has been induced using PGRs for the efficient production of secondary metabolites, which include *Morinda coreia* Buck. and Ham. [15], *Hypericum perforatum* [16], and *Eurycoma longifolia* [17].

In recent years, medium optimization, combined elicitor supplementation, and bioreactor application have been studied for high-yield secondary metabolites production. Elicitors are defined as any chemicals that trigger a mechanism in living organisms to produce secondary metabolites. Elicitors can be categorized into two types: biotic elicitors, including bacteria, viruses, and fungi; and abiotic elicitors, including metal ions and inorganic compounds (chemical elicitors), such as jasmonic acid (JA), methyl jasmonate (MeJA), salicylic acid (SA), acetyl salicylic acid (ASA), and nitric oxide (NO), which create a stress-like environment in a culture system of plants, triggering secondary metabolites production. For instance, the highest elicitation of anthraquinones (24.33 mg/g dry weight) was obtained from adventitious roots of *Oldenlandia* *umbellata* when 50 mg/L of pectin was added [18]. Additionally, phenolic and flavonoid contents were increased without affecting the adventitious root biomass in *Oplopanax elatus* when MeJA was added to the culture medium after 30 days of culture [19].

**Figure 1:**
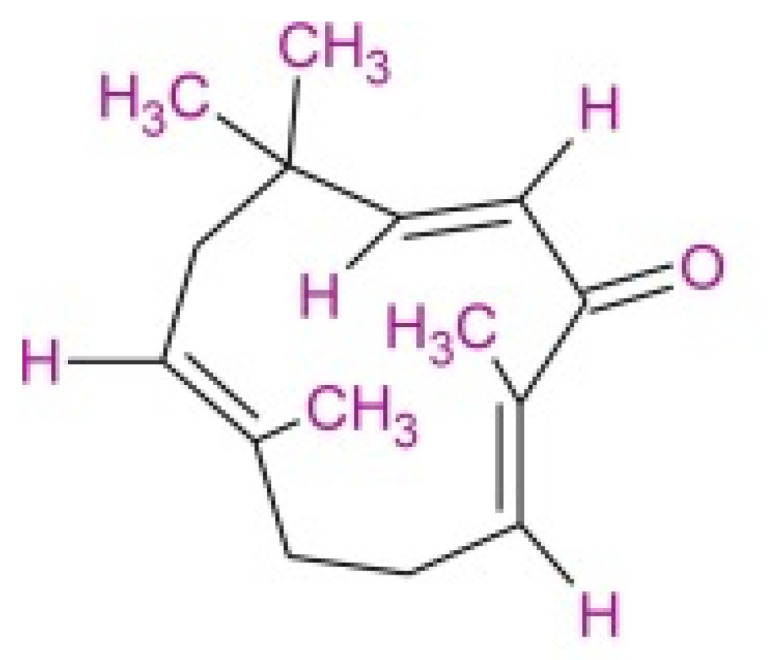
Chemical structure of zerumbone.

**Figure 2:**
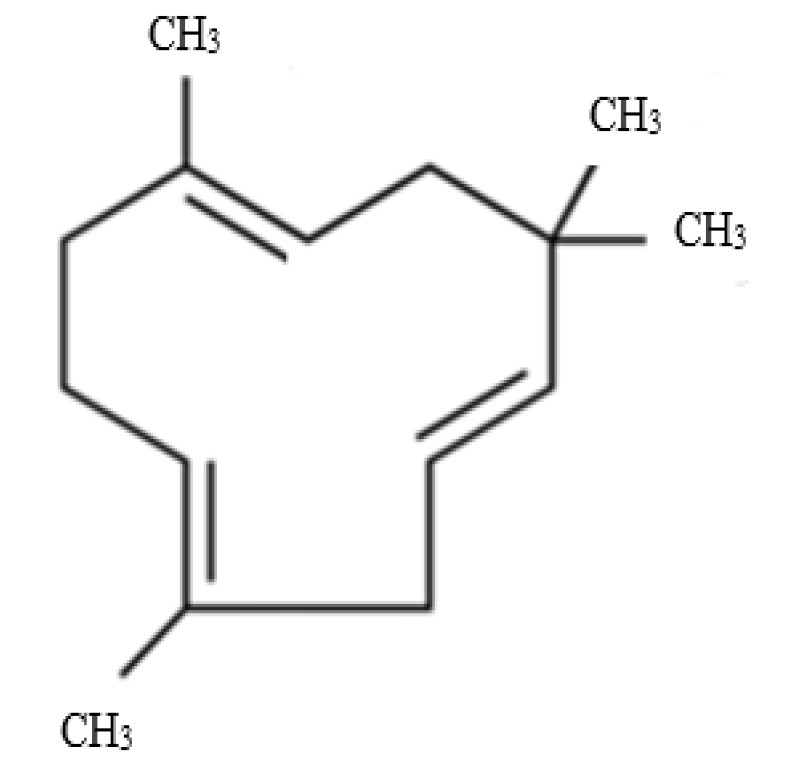
Chemical structure of α-humulene.

Zerumbone is a sesquiterpene that can be identified by three double bonds (Figure 1), and a double conjugated carbonyl group inside an 11-membrane ring structure. Zerumbone can be categorized as a polar and non-polar compound with a molecular weight of 218.3 g/mol. Several IUPAC names are identified for zerumbone, namely (E,E,E)-2,6,9,9-tetramethyl-2,6,10-cycloundecatrein-1-one, 2,6,10-cycloundecatrein-1-one, 2,6,9,9-tetramethyl-, and (E,E,E)-471-05-6. In addition, humulene, also known as α-humulene or α-caryophyllene, is a naturally occurring monocyclic sesquiterpene (C_15_H_24_) that contains an 11-membered ring, and consists of three isoprene units containing three non-conjugated C=C double bonds (Figure 2). The IUPAC name for this compound is 2,6,6,9-tetramethyl-1,4-8-cycloundecatriene, and it is also called α-caryophyllene or 3,7,10-humulatriene. The biosynthetic pathway of these compounds begins with the cyclization of (2*E*, 6*E*)-farnesyl diphosphate. In the pathway, the terpene synthase catalyzes the synthesis of α-humulene. Thereafter, α-humulene is converted to 8-hydroxy-α-humulene by α-humulene-10-hydroxylase, followed by conversion to zerumbone by zerumbone synthase. Therefore, the investigation focused on the enhancement of root culture growth, and the production of α-humulene and zerumbone in AdRC of *Z. zerumbet* Smith through the synergistic application of plant growth regulators and elicitors.

## 2. Materials and Methods

### 2.1. Plant Materials

Mature rhizomes of *Zingiber zerumbet* Smith (herbarium number 041148) were collected from a nursery at Pusat Asasi Sains, Universiti Malaya, in April 2018. They were cleaned thoroughly by repeated washing and maintained in the laboratory for sprouting.

### 2.2. Explant Sterilization

Young buds 1.5–2.0 cm in length were used as explants, and surface sterilized with 70% (*v*/*v*) Clorox (5.25% *w*/*w* sodium hypochlorite) solution with a few drops of Tween 20. After 30 min, the outer layer of the shoot buds was removed and transferred aseptically into 30% (*v*/*v*) Clorox for 10 min. Subsequently, the shoot buds were rinsed with sterile distilled water, and air-dried in a laminar air-flow chamber.

### 2.3. Initiation of Adventitious Root Culture of Z. zerumbet

The sterilized shoot buds were excised horizontally to 1.0 × 1.0 cm in length, and inoculated onto a petri dish containing Murashige and Skoog (1962) (MS) medium supplemented with a combination of naphthalene acetic acid (NAA) and indole-3-butyric acid (IBA) at a concentration of 0.5, 1, or 2 mg/L and 3% (*w*/*v*) sucrose. The media were solidified with 2 g/L gelrite. Explants grown in medium without hormones served as a control. The pH of the medium was adjusted to 5.7 prior to autoclaving. The cultures were maintained at 25 ± 1 °C under total dark conditions. For each treatment, the percentage of root response, root length, and the number of roots per explant were measured. All experiments were carried out in triplicate cultures with five explants in each replicate.

### 2.4. Combined Effects of Auxin and Cytokinin on Adventitious Root Growth and Secondary Metabolite Production of Z. zerumbet Root Suspension Culture

After one month of induction in an optimized media (1.0 mg/L NAA and 2.0 mg/L IBA), the induced roots were transferred into shake flasks containing different media formulations. The roots were cultured on MS media with different combinations of 6-benzylaminopurine (BAP) and indole-3-butyric acid (IBA) at concentrations of 1, 3, 5, and 7 mg/L, and supplemented with 3% *w*/*v* sucrose. Roots grown in a medium containing 1 mg/L NAA served as a control. The medium pH was adjusted to 5.7 prior to autoclaving. The root cultures were maintained at 25 ± 1 °C on an orbital shaker at 70 rpm   with a photoperiod of 16 h light (light intensity 31.4 μ mol/m^2^/s) and 8 h dark (total darkness). For each treatment, the fresh weight (FW) and dry weight (DW) were measured at six-day intervals over 30 days of culture. The growth parameter, namely the specific growth rate (μ), was determined for the AdRC by Equation (1). All experiments were carried out in triplicate. Roots were maintained in the optimized media from this experiment, and subculture every 30 days for further elicitation experiments.

(1) $$\mu =\frac{ln\left(\frac{x}{x_{0}}\right)}{∆t}$$

where

*μ* = Specific growth rate (day^−1^)

*x*_0_ = Initial fresh weight

*x* = Final fresh weight after incubation

Δ*t* = period of incubation (days)

### 2.5. Synergistic Effects of Elicitors on Root Growth and Bioactive Compounds Production

The root cultures were maintained at 25 ± 1 °C in orbital shaker at 70 rpm. Elicitation by different combinations of methyl jasmonate (MeJA) and salicylic acid (SA) at the followings were carried out: (T1) 400 µM MeJa + 400 µM SA; (T2) 400 µM MeJa + 600 µM SA; (T3) 400 µM MeJa + 800 µM SA; (T4) 400 µM MeJa + 1200 µM SA; (T5) 600 µM MeJa + 400 µM SA; (T6) 600 µM MeJa + 600 µM SA; (T7) 600 µM MeJa + 800 µM SA; (T8) 600 µM MeJa + 1200 µM SA; (T9) 800 µM MeJa + 400 µM SA; (T10) 800 µM MeJa + 600 µM SA; (T11) 800 µM MeJa + 800 µM SA; (T12) 800 µM MeJa and 1200 µM SA; (T13) 1200 µM MeJa + 400 µM SA; (T14) 1200 µM MeJa + 600 µM SA; (T15) 1200 µM MeJa + 800 µM SA; (T16) 1200 µM MeJa + 1200 µM SA.

The combination treatments were introduced on day 15 for a 26-day cultivation period. AdRC without elicitation was used as a control, and was harvested after 26 days. A stock solution of MeJA and SA was prepared separately by the dissolution of each substance in distilled water and filter sterilized using 0.22 μm filters prior to its addition to the root suspension cultures. Meanwhile, for the untreated sample, an equivalent volume of sterilized distilled water was added to the cultures as a blank treatment control for the different elicitor concentrations studied. All experiments were performed in triplicate, with each treatment repeated three times.

### 2.6. Bioactive Compound Extraction

After each treatment, roots were harvested and washed with distilled water followed by drying at 60 °C [20] for 24 h. Subsequently, 0.1 g of dried root was put into a cellulose extraction thimble for Soxhlet extraction. A water bath was set to 39 °C, corresponding to the boiling point of dichloromethane. The condenser temperature was set to 13–14 °C for a period of two hours. The same methods were used to obtain in vivo crude extract from rhizomes of *Z. zerumbet.*

### 2.7. Measurement of Zerumbone and α-Humulene

HPLC analysis was performed using a Shimadzu HPLC system, which consisted of SIL-20A HT autosampler, LC-20 AT multisolvent delivery system, DGU-20A degasser, CTO-20 AC cooler, and SPD-M20A UV/Visible detector, and Chromolith RP-18 endcapped column (100 × 4.6 mm) was associated with the LC solution software. HPLC-grade organic solvents, i.e., acetonitrile, methanol, and phosphoric acid (BDH), were used. The mobile phase consisted of a mixture of ultrapure water with 0.1% phosphoric acid (A) and acetonitrile (B). Analyses of zerumbone and α-humulene were carried out by the following elution gradient: 0.01 min, 50% A, 50% B; 2.00 min, 15% A, 85% B; 7.00 min, 15% A, 85% B; 7.50 min, 50% A, 50% B; 11.00 min, 60% A, 40% B. The total running time was 20 min at a flow rate of 1.0 mL/min. The temperature of the column was set at 35 °C at UV detection wavelength 243 nm. All samples were filtered through 0.25 µm membrane filters prior to injection into the HPLC. The zerumbone and α-humulene compounds were identified by matching the retention times and spectral characteristics of a sample to a commercially available standard (Sigma Aldrich, St. Louis, Missouri USA). Quantitative analysis was performed using the peak area calibrated to external standard concentration. Three replicates were prepared for each standard concentration. The injection was also performed in triplicate.

### 2.8. Statistical Analysis

Data were analyzed by one-way ANOVA followed by Tukey’s HSD test at a significance level of *p* < 0.05.

## 3. Results and Discussion

### 3.1. Initiation of Adventitious Root Culture (AdRC) of Z. zerumbet

**Figure 3:**
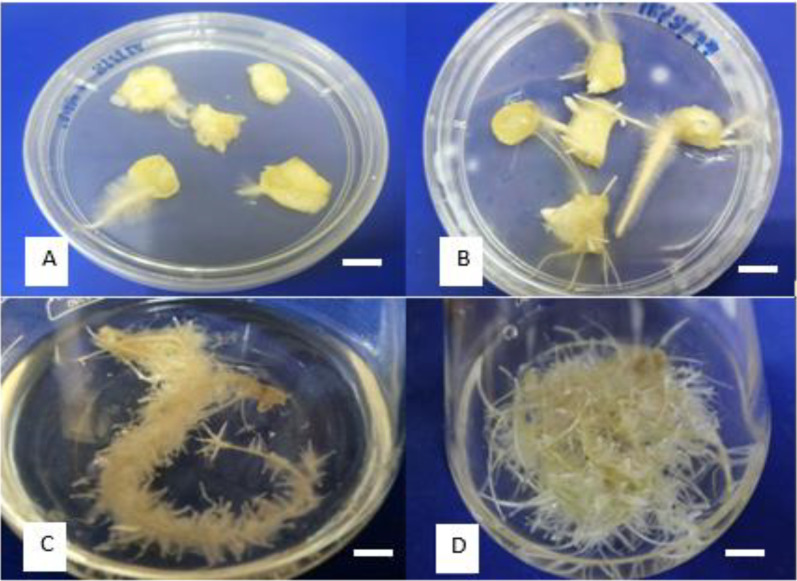
Establishment of AdRC of Z. *zerumbet.* (**A**) Root induction in control treatment; (**B**) root induction in MS medium supplemented with 1 mg/L NAA and 2 mg/L IBA; (**C**) multiplication of AdRC in MS medium supplemented with 1 mg/L NAA and 3 mg/L BAP after 5 days in shake flask; (**D**) multiplication of AdRC in MS medium supplemented with 1 mg/L NAA and 3 mg/L BAP after 15 days in shake flask. Bar: 0.5 cm.

AdRC is a viable route for secondary metabolite production due to its high proliferation rate and stable production of valuable secondary metabolites [21]. In this study, the root induction time varied with different concentrations and combinations of PGRs. The earliest adventitious root formation was observed seven days after inoculation in the MS medium supplemented with 1.0 mg/L NAA and 2.0 mg/L IBA, and incubated in the dark condition (Figure 3). Similar root emergence was observed in *Gynura procumbents*, which appeared after seven days of inoculation [22]. Even though the medium supplemented with 1.0 mg/L NAA and 1.0 mg/L IBA produced the highest number of roots (18.0 ± 1.0) with a rooting percentage of 93% (Table 1), media supplemented with 1.0 mg/L NAA and 2.0 mg/L IBA produced the highest number of root responses (100%), and numerous branching roots with a maximum root length of 7.3 ± 1.3 cm, as shown in Figure 3. Thus, the medium supplemented with 1.0 mg/L NAA and 2.0 mg/L IBA was selected as the optimal media for root induction, and roots induced from the formulation were used for further root suspension culture experiments.

### 3.2. Combined Effects of Auxin and Cytokinin on Adventitious Root Growth and Secondary Metabolite Production of Z. zerumbet Root Suspension Culture

A significant increase in adventitious root growth was observed with an increasing concentration of IBA up to 5 mg/L in both light regimes, contrary to the treatment combination with BAP (Table 2). The observation made was similar the cultivation of *Orthosiphon stamineus* [23], in which an increase in rooting efficiency was obtained with increasing IBA and NAA concentrations ranging from 1 mg/L to 5 mg/L. A combination of 0.5 mg/L NAA and 1.0 mg/L IBA enhanced the root growth of *Cichorium endivia* in total darkness was also reported [24]. It also corroborated the claim that a high IBA concentration promotes the development of malformed and thick roots whereas the combination of NAA with IBA provides a greater possibility to obtain high a rooting percentage in *Melissa officinalis* [25].

**Figure 4:**
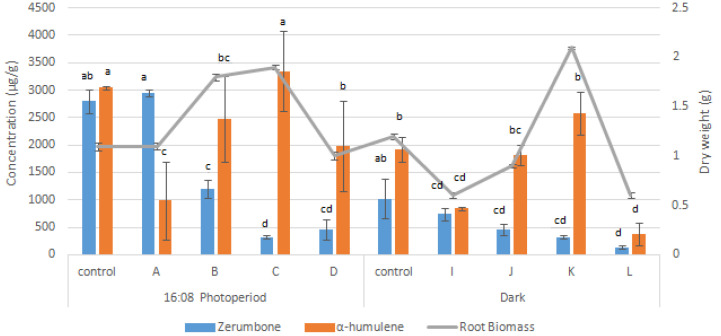
Zerumbone, α-humulene, and root dry weight produced in a combined auxin–auxin medium with a 16:08 photoperiod and dark condition. MS media supplemented with 1 mg/L NAA (control) combined with 1, 3, 5, and 7 mg/L IBA (treatments A, B, C, and D, respectively, with a 16:08 photoperiod) or with 1 mg/L NAA (control) combined with 1, 3, 5, and 7 mg/L IBA (treatments I, J, K, and L in the dark, respectively). Error bars indicate standard deviation of the mean value. Means with different letters in the same column are significantly different at *p* < 0.05 according to ANOVA and Tukey’s multiple range test.

**Figure 5:**
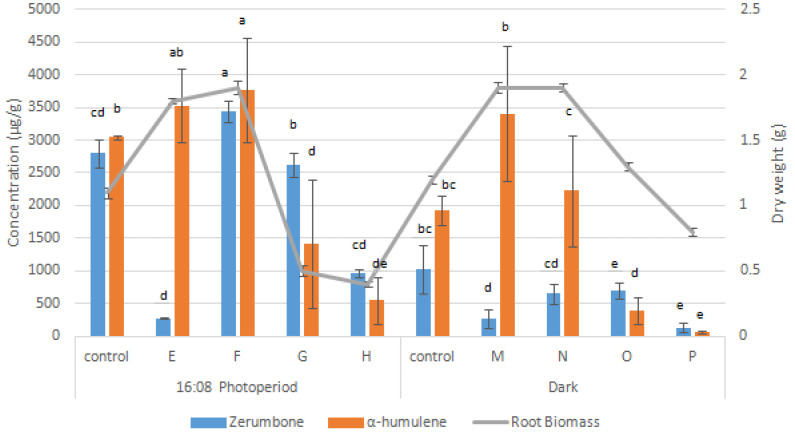
Zerumbone, α-humulene, and root dry weight produced in a combined auxin–cytokinin medium with a 16:08 photoperiod and dark condition. MS media supplemented with 1 mg/L NAA (control) combined with 1, 3, 5, and 7 mg/L BAP (treatments E, F, G, and H, respectively, with a 16:08 photoperiod) or with 1 mg/L NAA (control) added with 1, 3, 5 and 7 mg/L BAP (treatments M, N, O, and P in the dark, respectively). Error bars indicate standard deviation of the mean value. Means with different letters in the same column are significantly different at *p* < 0.05 according to ANOVA and Tukey’s multiple range test.

A new hormonal control method was suggested in which hormones should be introduced after root establishment whereby auxin should be at a lower concentration for root meristem maintenance, and cytokinin should be supplied for root tissue differentiation [26]. Treatment C (Table 2) produced the maximum fresh weight (6.9 ± 0.1 g FW) and dry weight (2.1 g DW) with the highest specific growth rate (2.1 ± 0.0 day^−1^). However, treatments F and K showed no significant difference when compared with treatment C in terms of FW, DW, and specific growth rate (Table 2). Nevertheless, treatments C and K produced significantly lower amounts of zerumbone (325 ± 34 µg/g) and α-humulene (3576 ± 388 µg/g) (Figure 4) than treatment F (Figure 5).

The treatment combination with the highest BAP and IBA (7 mg/L) concentrations (treatments G, O, P, and L) produced a significantly lower AdRC. In addition, the lowest specific growth rate and biomass of AdRC (Table 2) were observed at the highest concentrations of IBA and BAP (treatments D and H), as high concentrations of auxin and cytokinin can create a high stress condition for the biomass growth, which inhibits root formation. In terms of secondary metabolite production, the lowest titers of zerumbone (137 ± 70 µg/g) were obtained in treatment L, and the lowest titers of α-humulene (60 ± 22 µg/g) were obtained in treatment P (Figure 5). Conversely, in a study conducted on *Eurycoma longifolia,* higher concentrations of IBA and NAA ranging from 5 mg/L to 9 mg/L enhanced the accumulation of total phenolics and flavonoids [27].

Meanwhile, treatment F produced the highest levels of zerumbone (3440 ± 168 µg/g) and α-humulene (3759 ± 798 µg/g) with 1.90 ± 0.1 g of DW (Figure 5). Similarly, the maximum plumbagin production was observed in *Plumbago rosea* when it was cultivated in media containing auxin and cytokinin [28]. In addition, auxin downregulates the expression of genes associated with terpenoid indole alkaloid biosynthesis while cytokinin acts as an accelerant that enhances the accumulation of alkaloids in *Catharanthus roseus* root culture [29]. Thus, to ensure sustainable growth and compound yield, roots were maintained in the optimal media formulation (treatment F) for further elicitation experiments.

### 3.3. Synergistic Effects of Elicitors on Root Growth and Bioactive Compounds Production

**Figure 6:**
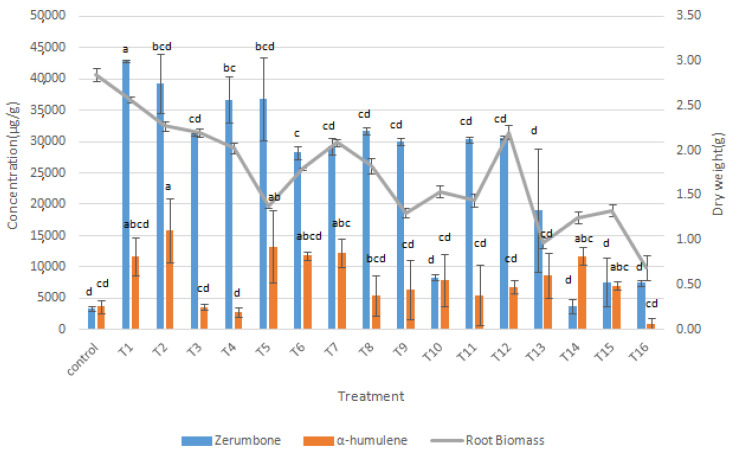
Synergistic effects of MeJa and SA on zerumbone, α-humulene, and root biomass of *Z. zerumbet* after 26 days of culture following elicitation: (T1) 400 µM MeJa + 400 µM SA; (T2) 400 µM MeJa + 600 µM SA; (T3) 400 µM MeJa + 800 µM SA; (T4) 400 µM MeJa + 1200 µM SA; (T5) 600 µM MeJa + 400 µM SA; (T6) 600 µM MeJa + 600 µM SA; (T7) 600 µM MeJa + 800 µM SA; (T8) 600 µM MeJa + 1200 µM SA; (T9) 800 µM MeJa + 400 µM SA; (T10) 800 µM MeJa + 600 µM SA; (T11) 800 µM MeJa + 800 µM SA; (T12) 800 µM MeJa and 1200 µM SA; (T13) 1200 µM MeJa + 400 µM SA; (T14) 1200 µM MeJa + 600 µM SA; (T15) 1200 µM MeJa + 800 µM SA; (T16) 1200 µM MeJa + 1200 µM SA. Error bars indicated standard deviation of the mean value. Means with different letters in the same column are significantly different at *p* < 0.05 according to ANOVA and Tukey’s multiple range test.

In this study, the root yield decreased with increasing concentrations of MeJa and SA, as shown in Figure 4. When the roots were treated with MeJa and SA at higher concentrations (600–1200 µM), the root biomass production decreased by 66% relative to the control for T15 (1200 µM MeJa + 800 µM SA). The reduction in root biomass following these treatments could be due to the inhibitory effects of the elicitors. Treatment with the highest concentration of elicitors (1200 µM MeJa + 1200 µM SA) yielded the lowest root biomass production at 0.69 g, i.e., 76% lower after elicitation than the control (Figure 6). This was similar to a study conducted on *Vicia faba*, where concentrations above 10 µM SA inhibited root growth in some species, while treatment with 5000 µM SA resulted in a 25% reduction [30].

However, the production of these compounds showed significant increase with treatments ranging from 400 µM to 800 µM of the elicitors’ combination compared to the control. Similarly, the combined treatment of MeJA and SA was found to enhance saponin production, which increased by 1.5- and 1.3-fold in the adventitious root culture of *Talinum paniculatum* (Javanese ginseng) [31].

While adding elicitors resulted in strong inhibition of root growth [32], the root biomass growth in the current study was not significantly inhibited at lower concentrations of combined elicitors. In addition, the amount of zerumbone was higher than that of α-humulene in all treated root cultures of *Z. zerumbet* (Figure 6). It is possible that the synergistic application of MeJa and SA triggered the plant defense system to produce more zerumbone than α-humulene. This was attributed to the upregulation of zerumbone synthase activity in the biosynthesis pathway during elicitation. The combined treatment of 400 µM MeJa + 400 µM SA yielded the highest improvement in zerumbone production (43,000 ± 0.2 µg/g), namely a 13-fold increase compared with the control. Meanwhile, treatment with 400 µM MeJa + 600 µM SA yielded the highest α-humulene production (15,800 ± 5.1 µg/g), namely a 4.3-fold increase compared with the control (Figure 6). Similarly, the synergistic effects of MeJa and SA at 0.1 mM increased the bilobalide and ginkgolide production in *Ginkgo biloba* cultures [33], which could be explained by the changes in its metabolomics pathways.

**Figure 7:**
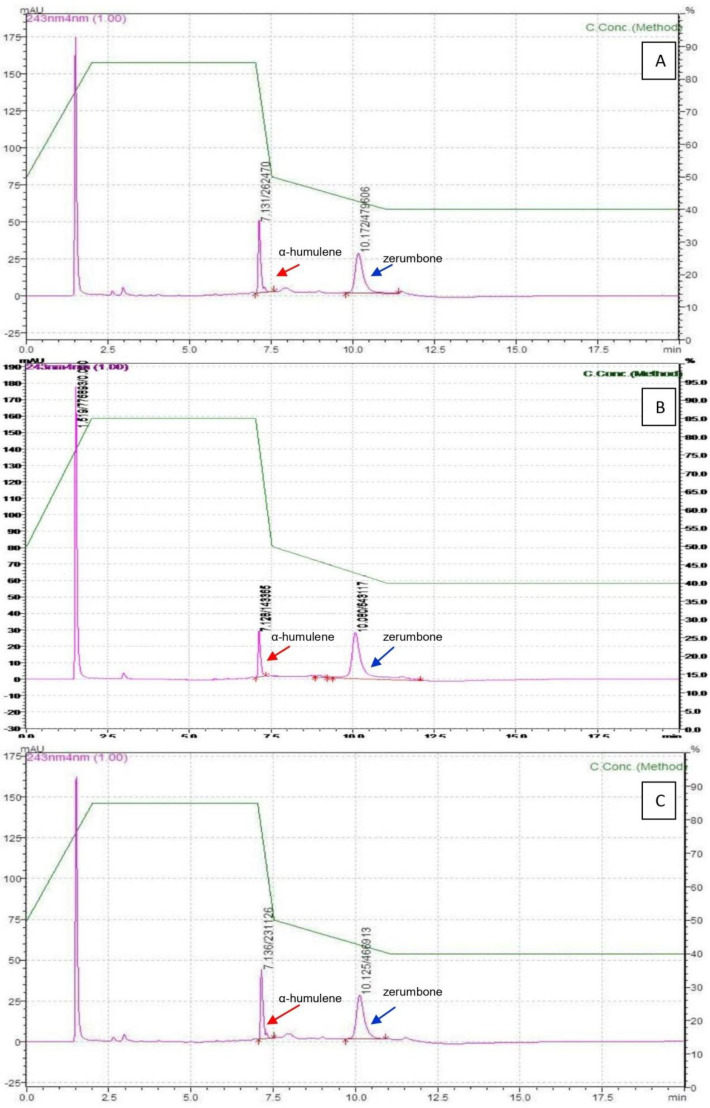
The HPLC chromatograms of *Z. zerumbet* samples. (**A**) AdRC elicited with 400 µM MeJa + 400 µM SA; (**B**) AdRC without elicitors; (**C**) rhizome.

The representative HPLC profile (Figure 7) showed a significant increase in zerumbone and α-humulene production when treated at a 400 µM concentration compared with the untreated control collected on the same day. The retention times for α-humulene and zerumbone were at 7.1 and 10.1 min, respectively, with a total run time of less than 20 min. No interfering peaks were observed. When compared with zerumbone and α-humulene present in the samples extracted from in vivo rhizomes, the increase in the elicited compounds ranged from 1- to 1.8-fold. Hence, the yields obtained from AdRC are comparable to zerumbone and α-humulene titers extracted from the rhizomes of field-grown plants.

## 4. Conclusions

The synergistic applications of PGR (auxin and cytokinin) and elicitors (methyl jasmonate and salicylic acid) at low concentrations enhanced zerumbone and α-humulene production relative to untreated control and in vivo rhizome of *Z. zerumbet* Smith. The elicitation of AdRC by 400 µM methyl jasmonate + 400 µM salicylic acid represents a successful method to enhance the productivity of α-humulene and zerumbone in *Z. zerumbet* AdRC. Further investigation of elicitor-induced metabolomic, and the synergistic application of elicitors in a large-scale system would help validate the findings of the current study and contribute to the natural drug development process.

## Figures and Tables

**Table 1:** Initiation of adventitious roots of *Z. zerumbet* in MS medium supplemented with different combinations and concentrations of plant growth regulators.

Plant Growth Regulators (mg/L)	Percentage (%)	Root Length (cm)	Roots per Explant
Control	13 ^de^	0.4 ± 0.1 ^e^	2.0 ± 0.6 ^e^
0.5 NAA + 0.5 IBA	20 ^d^	0.8 ± 0.4 ^ed^	1.6 ± 1.2 ^ef^
0.5 NAA + 1.0 IBA	60 ^b^	0.3 ± 0.1 ^e^	4.7 ± 1.5 ^cde^
0.5 NAA + 2.0 IBA	67 ^b^	2.1 ± 0.5 ^bcde^	6.7 ± 2.1 ^c^
1.0 NAA + 0.5 IBA	47 ^c^	1.7 ± 0.3 ^bcde^	4.7 ± 3.1 ^cde^
1.0 NAA + 1.0 IBA	93 ^a^	3.3 ± 0.7 ^b^	18.0 ± 1.0 ^a^
1.0 NAA + 2.0 IBA	100 ^a^	7.3 ± 1.3 ^a^	11.7 ± 1.5 ^b^
2.0 NAA + 0.5 IBA	40 ^c^	2.6 ± 0.8 ^bc^	5.7 ± 2.1 ^cd^
2.0 NAA + 1.0 IBA	73 ^b^	2.2 ± 0.3 ^bcd^	6.3 ± 1.5 ^c^
2.0 NAA + 2.0 IBA	87 ^a^	1.0 ± 0.6 ^cde^	5.3 ± 2.1 ^cd^

Means with different letters in the same column are significantly different at *p* < 0.05 according to ANOVA and Tukey’s multiple range test.

**Table 2:** Combined effects of auxin–auxin and auxin–cytokinin on adventitious root growth of *Z. zerumbet* in 16:08 photoperiod and dark condition.

Light Regime	PGR	Treatment	Concentration (mg/L)	Fresh Weight (g)	Dry Weight (g)	Specific Growth Rate (µ), Day^−1^
16:08	Control (NAA)	Control 16:08	1	3.9 ± 0.0 ^e^	1.2 ± 0.0 ^b^	1.4 ± 0.0 ^c^
	Control + IBA	A	1	2.0 ± 0.1 ^h^	0.6 ± 0.0 ^c^	0.8 ± 0.0 ^d^
		B	3	3.0 ± 0.1 ^g^	0.9 ± 0.0 ^bc^	1.2 ± 0.0 ^cd^
		C	5	6.9 ± 0.1 ^a^	2.1 ± 0.0 ^a^	2.1 ± 0.0 ^a^
		D	7	1.9 ± 0.1 ^i^	0.6 ± 0.0 ^c^	0.7 ± 0.0 ^d^
	Control + BAP	E	1	5.9 ± 0.1 ^c^	1.8 ± 0.0 ^ab^	1.9 ± 0.0 ^a^
		F	3	6.8 ± 0.1 ^a^	1.9 ± 0.1 ^a^	2.0 ± 0.1 ^a^
		G	5	1.8 ± 0.1 ^i^	0.5 ± 0.0 ^c^	0.7 ± 0.0 ^d^
		H	7	1.6 ± 0.1 ^i^	0.4 ± 0.0 ^c^	0.5 ± 0.0 ^e^
Dark	Control (NAA)	Control dark	1	3.6 ± 0.1 ^f^	1.1 ± 0.0 ^b^	1.4 ± 0.0 ^c^
	Control + IBA	I	1	3.6 ± 0.1 ^f^	1.1 ± 0.0 ^b^	1.4 ± 0.0 ^c^
		J	3	6.3 ± 0.1 ^bc^	1.9 ± 0.0 ^a^	1.9 ± 0.0 ^a^
		K	5	6.7 ± 0.1 ^a^	2.0 ± 0.0 ^a^	2.0 ± 0.0 ^a^
		L	7	3.4 ± 0.1 ^fg^	1.0 ± 0.0 ^b^	1.3 ± 0.0 ^c^
	Control + BAP	M	1	6.2 ± 0.0 ^bc^	1.9 ± 0.0 ^a^	1.9 ± 0.0 ^a^
		N	3	6.4 ± 0.1 ^b^	1.9 ± 0.0 ^a^	1.9 ± 0.0 ^a^
		O	5	4.4 ± 0.1 ^d^	1.3 ± 0.0 ^b^	1.6 ± 0.0 ^b^
		P	7	2.4 ± 0.1 ^h^	0.8 ± 0.0 ^c^	1.0 ± 0.0 ^cd^

Means with different letters in the same column are significantly different at *p* < 0.05 according to ANOVA and Tukey’s multiple range test.

## Data Availability

The datasets generated during and/or analyzed during the current study are not publicly available due to the university’s policy but are available from the corresponding author upon reasonable request.
